# Effects of some surface active ionic liquids on the aqueous solubility, thermodynamic properties and fluorescence behavior of drug diclofenac sodium

**DOI:** 10.1038/s41598-026-56699-9

**Published:** 2026-06-14

**Authors:** Mohammad Bagheri Hokm Abad, Hemayat Shekaari, Elaheh Janbezar, Shima Ghasemzadeh

**Affiliations:** Department of Physical Chemistry, Faculty of Chemistry, University of Tabriz, Tabriz, 5166616471 Iran

**Keywords:** Fluorescence quenching, Solubility, Surface active ionic liquid, Diclofenac sodium, Spectroscopy, COSMO, Chemistry, Materials science

## Abstract

Bio-based surface-active ionic liquids (SAILs) hold considerable promise for pharmaceutical applications, particularly in enhancing the aqueous solubility and bioavailability of poorly water-soluble drugs, such as diclofenac sodium (DFS), a nonsteroidal anti-inflammatory agent classified as BCS Class II. Three distinct (2-hydroxyethyl) amine-based surface-active ionic liquids (SAILs) were synthesized. Subsequently, the interactions between DFS and these synthesized SAILs were investigated using fluorescence spectroscopy at a temperature of 298.15 K. Fluorescence spectroscopy revealed a strong interaction between DFS and SAILs. This was evident from the significant quenching of DFS’s intrinsic fluorescence upon SAIL addition. The association constant and binding sites were determined. Among the tested SAILs, the [2-HEA][Ole] exhibited the strongest interaction with DFS. Furthermore, the solubility of DFS in aqueous SAILs solutions studied at temperature range of (298.15 to 313.15) K was found to increase with increasing SAIL concentration. The solubility data were accurately fitted using the *e*-NRTL and Wilson models. To gain deeper insights, conductor-like screening model (COSMO) calculations were performed on the studied chemicals. The obtained surface cavity volume (*V*) and dielectric solvation energy from the COSMO calculations provided valuable information about the intermolecular interactions. Finally, thermodynamic analysis using Gibbs and van’t Hoff equations indicated that the dissolution of DFS in these systems is an endothermic process.

**Supplementary Information:**

The online version contains supplementary material available at https://doi.org/10.1038/s41598-026-56699-9.

## Introduction

 Diclofenac sodium (DFS) is one of the most convenient prescribed non‑steroidal anti‑inflammatory drugs (NSAIDs), but its therapeutic effectiveness is limited by poor aqueous solubility and low bioavailability, which prevents formulation efficiency and often require extra solubilizer agents^1^. An advancement from environmentally sustainable aspect and pharmaceutically compatible solubilization media is critical for enhancing patient safety and promoting sustainability in pharmaceutical manufacturing. Existing approaches, including topical patches, sustained-release matrices, and microcapsule-based delivery systems, have reduced gastrointestinal, cardiovascular, and renal effects while improving effectiveness^2–4^. Ionic liquids (ILs) such as N ‑acetyl amino acid with cholinium‑based cation are biocompatible materials that utilizing them have increased the solubility of diclofenac sodium up to fourfold in aqueous media due to extensive hydrogen bonding, ion pairing, and π‑π interactions between drug molecules and ionic liquid cation and anion^5,6^. Ethanolamine‑based ionic liquids have attracted considerable attention due to their tunable physicochemical properties and their potential as more environmentally benign alternatives to conventional ionic liquids. However, their toxicity depends on factors such as chemical structure and concentration, and several studies have reported that certain ethanolamine‑derived ionic liquids exhibit relatively lower toxicity and improved biodegradability compared with many traditional ionic liquids^7,8^. These ionic liquids can also be utilized into advanced dosage systems such as spray‑encapsulated solids and transdermal gels for controlled and localized delivery of diclofenac sodium^9^.

The mechanistic insights have been investigated from spectroscopic and thermodynamic approaches that indicates how ILs alter ionization equilibria, micellization, and electrostatic screening of drug molecules. Cholinium‑based ionic liquids and hydroxyl‑functionalized ILs have improved the concept of biocompatibility and low toxicity, highlighting the important role of hydrogen bonding in stabilizing drug with IL complexes^10,11^. Ethanolamine‑based ionic liquids provide a versatile, sustainable alternative to conventional solvents for improving drugs such as diclofenac sodium solubility, enabling safer formulations with enhanced bioavailability and therapeutic efficacy. Additionally, ethanolamine‑based ILs, namely (2-hydroxyethyl) ammonium oleate, bis(2-hydroxyethyl) ammonium oleate, and tris(2-hydroxyethyl) ammonium oleate represent a novel category of protic, amphiphilic solvents combining hydroxyethyl amine cations with long‑chain oleate anions. This synergistic configuration provides hydrophilic hydrogen‑bond donors and hydrophobic stabilization zones capable of dispersing diclofenac sodium ions. Imidazolium based ionic liquids are toxic but, these systems are synthesized from renewable and biodegradable precursors, yielding an intrinsically green material for solubilization studies. Their structural adjustability allows rational tuning of polarity and cohesive energy density, making them ideal candidates for controlled equilibrium investigations^12,13^.

In this study, the solubility enhancement of diclofenac sodium (DFS) was studied using hydroxyl-functionalized ammonium oleate SAILs, ([2-HEA][Ole], [BHEA][Ole], [THEA][Ole]) SAILs under solid–liquid equilibrium (SLE) at temperatures of (298.15 to 313.15) K. The concentration of the dissolved diclofenac sodium was determined using fluorescence spectroscopy, which allowed examination of molecular-level interactions between DFS and SAILs. This spectroscopic technique yielded quantitative data regarding the specific quenching mechanisms, association constants, and stoichiometry of the binding sites, elucidating the self-assembly of stable complexes between DFS and the investigated SAILs. The experimental solubility data were subsequently correlated employing thermodynamic models, including the Wilson and electrolyte non-random two-liquid (*e*-NRTL) equations, to characterize the non-ideal behavior of the systems and elucidate solute–solvent interactions. Temperature-dependent solubility profiles were analyzed using the van’t Hoff and Gibbs equations to derive the changes in enthalpy (Δ*H*), entropy (Δ*S*), and Gibbs free energy (Δ*G*) associated with dissolution, thereby identifying the dominant thermodynamic driving forces. Complementary density functional theory (DFT) calculations, implemented via the DMol³ module with conductor-like screening model (COSMO) solvation, were conducted to define and quantify the electronic interactions within the DFS–SAILs assemblies. Through targeted computational workflows, we successfully mapped essential structural and electronic indices, including localized solvation energies, molecular surface cavity areas (A), the highest and lowest unoccupied molecular orbitals (HOMO and LUMO), and molecular cavity volumes (*V*). These results can assist us in thoroughly exploring the microenvironmental elements controlling the drug’s improved solubility profiles. Notably, whereas semi-empirical thermodynamic equations such as Wilson, e-NRTL, and van 't Hoff serve as standards for mass-transfer correlations, there is a void in the literature about their application to multi-component drug solubilization through bio-inspired SAILs. Crucially, comprehensive study on the precise structural function of oleate-based SAILs functionalized with hydroxyethylamine cations in modifying the solvation thermodynamics and spatial organization of diclofenac sodium has not yet been conducted.This work uses an integrated, cross-disciplinary methodology to directly address this gap in knowledge.Through the integration of macroscopic thermodynamic modeling, molecular fluorescence spectroscopy, empirical phase mapping, and quantum chemical calculations, we provide a comprehensive benchmarking approach for evaluating how sustainable, biocompatible ionic media can optimize the physicochemical delivery profiles of challenging amphiphilic therapeutics.

## Materials and methods

A detailed characterization of the materials employed in this study is presented in Table 1, showcasing their chemical formulas, provenance (sources), molar masses, purities, and CAS registry numbers. The deionized double-distilled water utilized in all experiments exhibited a specific electrical conductance of less than 1 µS cm⁻¹. All experiments were conducted at ambient laboratory pressure. The uncertainties associated with the experimental measurements carried in this study were evaluated according to the guidelines provided by the National Institute of Standards and Technology (NIST). All measurements were performed in triplicate (*n* = 3), and the reported uncertainties represent the standard deviation of the measurements. The experimental deviations were very small; therefore, error bars are not visible on the plots to preserve figure clarity.

**Table 1:** Description of the utilized materials ^***a***^.

Materials name	Chemical formula	Provenance (Source)	CAS.no	Molar mass (g mol^− 1^)	purity
2-Hydroxyethylamine or monoethanolamine	C_2_H_7_NO	Merck	141-43-5	61.08	> 99%
Bis(2-hydroxyethyl) amine or diethanolamine	C_4_H_11_NO_2_	Merck	111-42-2	105.14	> 99%
Tris(2-hydroxyethyl) amine or triethanolamine	C_6_H_15_NO_3_	Merck	102-71-6	149.19	> 99%
Oleic acid	C_18_H_34_O_2_	Merck	112-80-1	318.13	> 99%
Diclofenac sodium	C_14_H_10_C_l2_NNaO_2_	Merck	15307-79-6	296.15	> 99%
(2-Hydroxyethyl)ammonium oleate [2-HEA][Ole]	C_20_H_41_NO_3_	Synthesized	–	343.55	> 98%
Bis(2-hydroxyethyl)ammonium oleate [BHEA][Ole]	C_22_H_45_NO_4_	Synthesized	–	387.33	> 98%
Tris(2-hydroxyethyl)ammonium oleate [THEA][Ole]	C_24_H_49_NO_5_	Synthesized	–	431.66	> 98%

a All of the attained chemicals from the supplier were used without further purification.

### Synthesis of surface-active ionic liquids (SAILs)

The surface-active ionic liquids (SAILs), namely (2-hydroxyethyl)ammonium oleate ([2-HEA][Ole]), bis(2-hydroxyethyl)ammonium oleate ([BHEA][Ole]), and tris(2-hydroxyethyl)ammonium oleate ([THEA][Ole]), were prepared via acid–base neutralization reactions. This methodology entailed the stoichiometric (1:1 molar ratio) combination of oleic acid with the corresponding ethanolamine derivatives monoethanolamine (MEA), diethanolamine (DEA), and triethanolamine (TEA)^14^. In a typical procedure, oleic acid was introduced into a reaction flask and heated to (323 to333) K under continuous magnetic stirring until a homogeneous melt was obtained. The requisite ethanolamine was then added dropwise while maintaining vigorous stirring at the elevated temperature. The physicochemical properties of the resultant SAILs were markedly influenced by the parent ethanolamine: [2-HEA][Ole] exhibited moderate viscosity, [BHEA][Ole] displayed elevated viscosity coupled with pronounced surface-active behavior, and [THEA][Ole] demonstrated superior emulsification efficacy^15–20^.

**Fig. 1:**
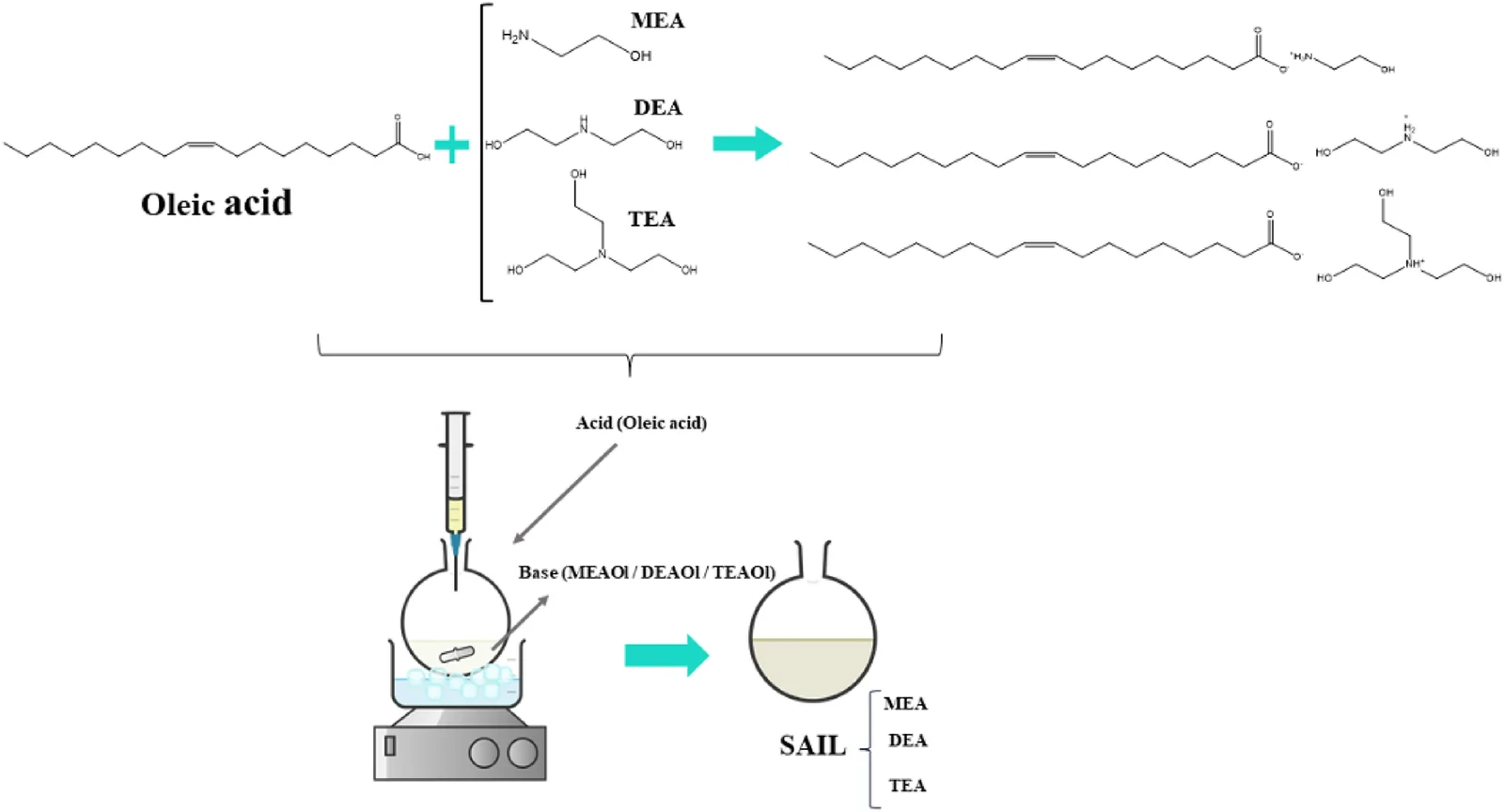
The synthesize route of SAILs.

The exothermic neutralization reaction required careful temperature control, maintaining temperatures below 343 K to prevent overheating. Observable changes during the reaction included a color shift from yellow to light brown and a marked increase in viscosity. After complete ethanolamine addition, the mixture was stirred at (333 to343) K for (2 to 4) h to ensure reaction completion^21^. The product was then vacuum dried and heated at (313 to323) K to remove residual solvents. The FT‑IR spectra and ¹H NMR chemical shift assignments are consistent with the expected functional groups and structural features of the synthesized SAILs (presented in Figures S2–S7). No additional signals corresponding to major impurities were observed within the detection limits of the measurements. The synthesized SAILs demonstrated distinct viscosity, surface activity, and thermal stability, supporting their potential use in detergents, petroleum, pharmaceuticals, and agriculture.

The critical micelle concentration (CMC) values of the investigated SAILs were determined in our previous studies by Janbezar et al.^22,23^ to be 0.00930 mol/kg for [THEA][Ole], 0.0111 mol/kg for [BHEA][Ole], and 0.0115 mol/kg for [2‑HEA][Ole]. The SAIL concentrations employed in the present study are comparable to or higher than these CMC values, indicating that micelle‑like aggregates are likely present in solution and may contribute to the observed solubilization behavior.

### Sample preparation

Stock solutions of surface-active ionic liquids (SAILs) in aqueous diclofenac sodium were prepared at varying concentrations using an analytical balance (model AND GR220; precision ± 1 × 10^− 8^ kg).

### Solubility determination

The solubility of DFS in diverse (2-hydroxyethyl) ammonium-based SAILs was quantified employing the saturation shake-flask method, a technique esteemed for its precision and reproducibility in physicochemical determinations^24^. Solubility measurements were performed over a temperature range of (298.15 to 313.15) K, with precise thermal regulation achieved using a thermostatically controlled water bath (model ED, Julabo GmbH, Seelbach, Germany) exhibiting a temperature stability of ± 0.01 K. For each determination, an accurately weighed aliquot of the solvent mixture (comprising the surface-active ionic liquid (SAIL) and water) was transferred to sealed glass vials, followed by the introduction of excess diclofenac sodium (DFS) to establish supersaturation. Vigorous agitation was induced using a Behdad orbital shaker at a temperature exceeding the target value by 5 K for 24 h to promote thorough homogenization. The resultant solid–liquid suspensions were subsequently transferred to the water bath and maintained quiescent for 72 h to attain thermodynamic equilibrium. Following equilibration, the samples were allowed an additional 7 h for complete phase disengagement. The supernatant saturated solutions were then isolated by filtration through 0.22 μm polytetrafluoroethylene (PTFE) syringe filters (Whatman, Maidstone, UK) or 0.45 μm hydrophilic Durapore^®^ polyvinylidene fluoride (PVDF) membrane filters (Millipore, Bedford, MA, USA) to eliminate residual undissolved solute. Preliminary adsorption experiments verified negligible DFS sorption onto the filtration media (< 0.5% rlative loss). The clarified filtrates were appropriately diluted with a water–ethanol binary mixture (2:8 v/v) prior to quantitative analysis. The DFS concentration in these diluted aliquots was quantified spectrophotometrically using a T80 + UV–vis double-beam spectrophotometer (PG Instruments Ltd., Lutterworth, UK) at the characteristic absorption maximum of 265 nm. A calibration plot (Fig. 1), constructed with water as the blank solvent, yielded a linear response with a correlation coefficient (R²) of 0.9986, thereby affirming the method’s robustness and precision.

**Fig. 2:**
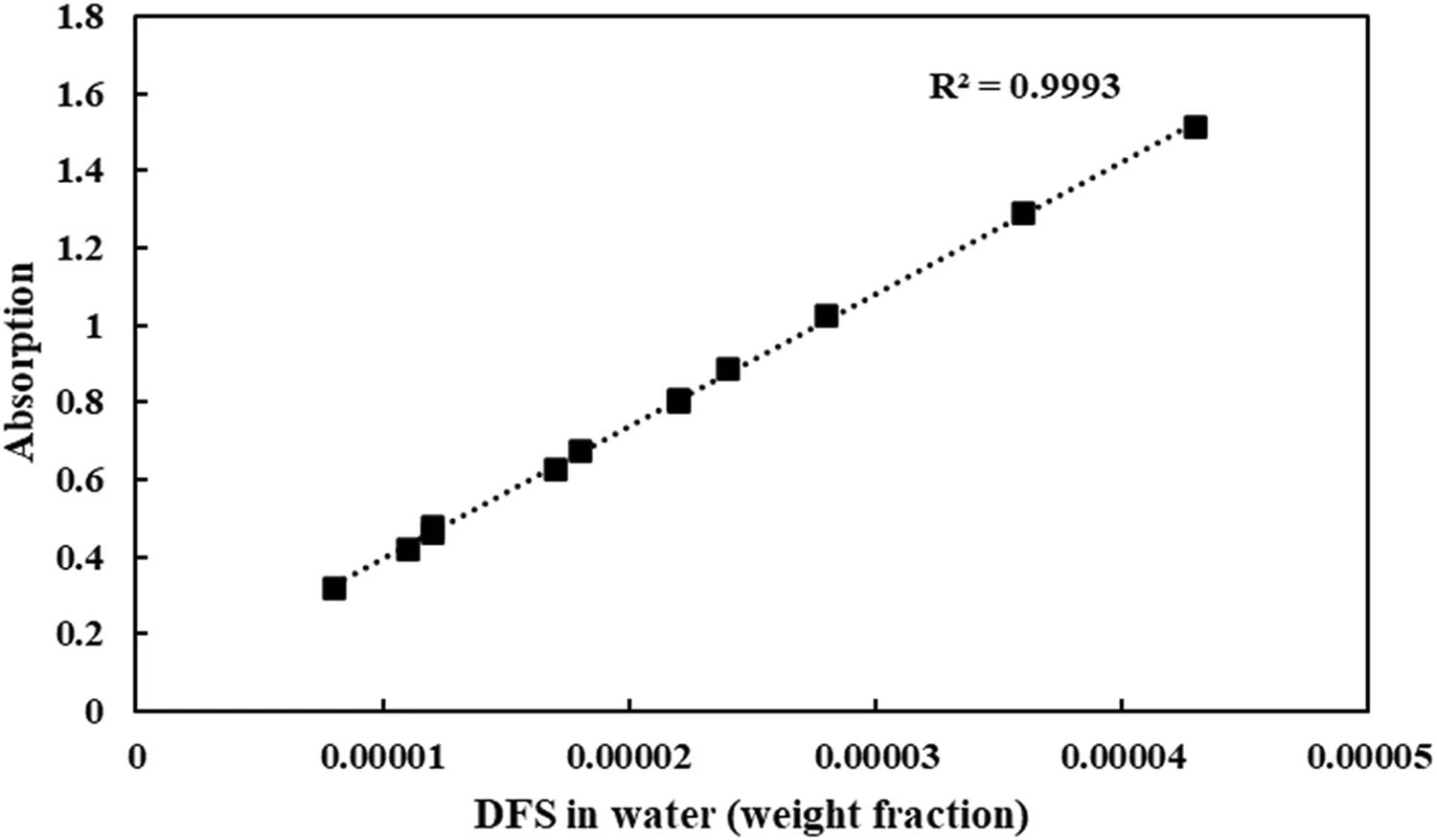
The calibration curve of the DFS.

The absorption maximum (λ_max_) of DFS was recorded at 276 nm, with no discernible bathochromic or hypochromic shifts observed in the presence of the SAILs. Complementary UV–vis spectrophotometric investigations further substantiated the lack of spectral overlap or interference from the SAILs on the characteristic absorbance of DFS.

The mole fraction solubility (*x*_1_) of DFS at a given temperature was computed according to the following expression:

1 $$x_{1} = \frac{{\frac{{w_{1} }}{{M_{1} }}}}{{\frac{{w_{1} }}{{M_{1} }} + \frac{{w_{2} }}{{M_{2} }} + \frac{{w_{3} }}{{M_{3} }}}}$$

where *M*_1_, *M*_2_, *M*_3_ are the molar mass of DFS, water, and SAIL respectively. Also, *w*_1_, *w*_2_, *w*_3_ represent the weight fractions of the DFS, water, and SAILs in the saturated solutions, respectively.

### Fluorescence spectroscopic measurements

Aqueous mixtures comprising DFS and SAILs were prepared by combining the respective stock solutions to yield a series of defined concentrations. The DFS concentration was maintained constant at 300 µm (10^− 6^ mol kg^− 1^), while the SAIL concentrations spanned (370, 550, 634, 880, 1100, 1460, 1820, and 2350) µm. For fluorescence spectroscopic analysis, each DFS–SAIL mixture was equilibrated in a thermostatically controlled water bath at 298.15 K for 4 h. Emission spectra of DFS in the aqueous SAIL media were recorded on a Hitachi F‑4500 fluorescence spectrophotometer (Tokyo, Japan) using an excitation wavelength (λex) of 260 nm. The emission spectra (λem) were collected over the range of 265–625 nm, with a photomultiplier tube voltage of 700 V and excitation and emission slit widths of 10 nm.

## Results and discussion

### Solubility results

The solubility of DFS in aqueous surface-active ionic liquid (SAIL) mixtures over the examined temperature range has been expressed on a mole fraction basis. It is noteworthy that the data delineated in Table 2 evince a consistent solubility profile across the investigated systems.

**Table 2:** Experimental (*x*_1_^exp^)^a^ and calculated ( *x*_1_^cal^) solubility mole fraction of DFS in the aqueous SAIL solutions at different temperatures (*T*)^b^ and weight fractions of SAILs (*w*_*3*_ )^c^ from *e*-NRTL and Wilson models.

T (K)		e-NRTL model		Wilson model	
	10^4^ x_1_^exp^	10^4^ x_1_^cal^	100 × (x_1_^exp^ -x_1_^exp^)/x_1_^exp^	10^4^ x_1_^cal^	100 × (x_1_^exp^ -x_1_^exp^)/x_1_^exp^
DFS (1) + Water (2) + [2-HEA] [Ole] (3)					
^*^w_3_ = 0.0000					
298.15	0.789	0.790	-0.03	0.790	0.02
303.15	0.831	0.817	1.71	0.830	0.06
308.15	0.879	0.890	-1.26	0.878	0.01
313.15	0.918	0.916	0.25	0.918	0.03
*w*_3_=0.1000					
298.15	21.602	21.055	2.53	21.585	0.08
303.15	22.224	21.899	1.46	22.203	0.09
308.15	26.400	25.556	3.2	26.336	0.05
313.15	26.630	25.935	1.76	26.616	0.25
*w*_3_=0.3000					
298.15	41.232	37.964	7.93	41.257	-0.06
303.15	41.452	39.167	5.51	41.446	0.02
308.15	41.740	40.583	2.77	41.779	-0.09
313.15	46.541	43.109	7.37	46.583	-0.09
*w*_3_=0.5000					
298.15	66.445	61.954	6.76	66.444	0.01
303.15	70.060	65.205	6.93	70.009	0.07
308.15	74.532	68.141	8.58	74.595	-0.04
313.15	76.999	73.365	4.72	77.030	-0.08
298.15	0.789	0.790	-0.03	0.790	0.02
303.15	0.831	0.817	1.71	0.830	0.06
308.15	0.879	0.890	-1.26	0.878	0.01
313.15	0.918	0.916	0.25	0.918	0.03
*w*_3_=0.7000					
298.15	128.711	114.569	10.99	128.275	0.34
303.15	145.157	133.082	8.32	144.737	0.29
308.15	146.318	132.803	9.24	144.950	0.93
313.15	152.131	139.432	8.35	152.000	0.09
*w*_3_=0.9000					
298.15	317.720	294.101	7.43	319.343	-0.51
303.15	358.173	332.669	7.12	366.012	-2.19
308.15	363.650	340.019	6.5	359.875	1.04
313.15	371.778	346.617	6.77	371.013	0.21
*w*_3_=1.0000					
298.15	659.940	671.264	-1.72	657.788	0.33
303.15	671.013	665.110	0.88	667.714	0.49
308.15	708.304	708.189	0.02	712.977	-0.66
313.15	747.646	737.608	1.34	748.421	-0.1
DFS (1) + Water (2) + [BHEA][Ole] (3)					
*w*_3_=0.1000					
298.15	12.43	12.310	0.99	12.378	0.44
303.15	12.981	12.522	3.54	12.900	0.63
308.15	13.294	12.769	3.95	13.319	-0.19
313.15	14.055	13.521	3.81	14.063	-0.05
*w*_3_=0.3000					
298.15	28.720	27.355	4.75	28.638	0.28
303.15	30.716	29.561	3.76	30.500	0.70
308.15	30.681	29.496	3.86	30.596	0.28
313.15	31.760	30.493	3.99	31.816	-0.18
*w*_3_=0.5000					
298.15	63.544	57.869	8.93	63.320	-0.35
303.15	74.686	65.893	11.77	74.176	0.68
308.15	75.014	65.988	12.03	74.538	0.63
313.15	75.576	67.359	10.87	75.643	-0.09
*w*_3_=0.7000					
298.15	110.105	99.225	9.88	110.004	0.09
303.15	114.613	104.637	8.7	114.487	0.11
308.15	118.059	108.766	7.26	108.035	0.08
313.15	113.714	109.354	9.99	113.592	0.02
*w*_3_=0.9000					
298.15	228.379	217.586	4.73	228.374	0.01
303.15	236.138	217.586	3.56	235.945	0.08
308.15	243.371	228.234	6.22	242.865	-0.02
313.15	285.384	265.559	6.95	285.447	0.21
*w*_3_=1.0000					
298.15	387.420	393.574	-1.59	387.530	-0.03
303.15	411.048	431.082	-4.87	410.880	0.04
308.15	443.621	436.380	1.63	443.718	-0.02
313.15	474.950	462.928	2.53	475.029	-0.02
DFS (1) + Water (2) + [THEA][Ole] (3)					
*w*_3_=0.01000					
298.15	5.076	4.267	15.93	5.099	-0.47
303.15	5.510	4.757	13.64	5.521	-0.23
308.15	7.250	6.438	11.20	7.254	-0.02
313.15	7.770	6.539	15.84	7.770	-0.01
*w*_3_=0.3000					
298.15	6.817	6.817	2.31	6.820	-0.04
303.15	7.281	6.748	7.33	6.748	0.30
308.15	8.027	7.251	9.67	8.009	0.22
313.15	8.407	7.605	9.54	8.407	0.04
*w*_3_=0.5000					
298.15	10.677	10.206	4.41	10.612	0.61
303.15	10.826	10.347	4.42	10.767	0.55
308.15	11.445	11.216	2.07	11.440	0.11
313.15	15.445	15.426	5.33	15.426	0.19
*w*_3_=0.7000					
298.15	10.879	11.367	-4.49	10.917	-0.35
303.15	15.830	15.349	3.03	15.821	0.05
308.15	22.789	22.422	1.60	22.780	0.03
313.15	29.943	29.157	2.62	29.917	0.08
*w*_3_=0.9000					
298.15	36.865	36.249	1.67	36.855	0.03
303.15	53.673	52.616	1.97	53.550	0.23
308.15	58.573	61.216	-4.51	58.580	-0.01
313.15	76.906	76.561	0.45	76.832	0.10
*w*_3_=1.0000					
298.15	207.104	212.274	-2.50	207.240	-0.07
303.15	216.172	213.080	1.40	216.071	0.01
308.15	221.399	231.249	-4.50	221.261	-0.01
313.15	226.451	227.754	-0.58	226.480	0.02

^*a*^ Standard uncertainty of experimental solubility is *u* ($x_{1}^{{\exp }}$) = 0.07.

^*b*^ Standard uncertainty of temperature is *u*(T) = 0.01 K.

^*c*^ Standard uncertainty of mass fraction is *u*(w_3_) = 0.0005.

^*^ The solubility of DFS in pure water is provided as a reference value and is not repeated in subsequent tables.

**Fig. 3:**
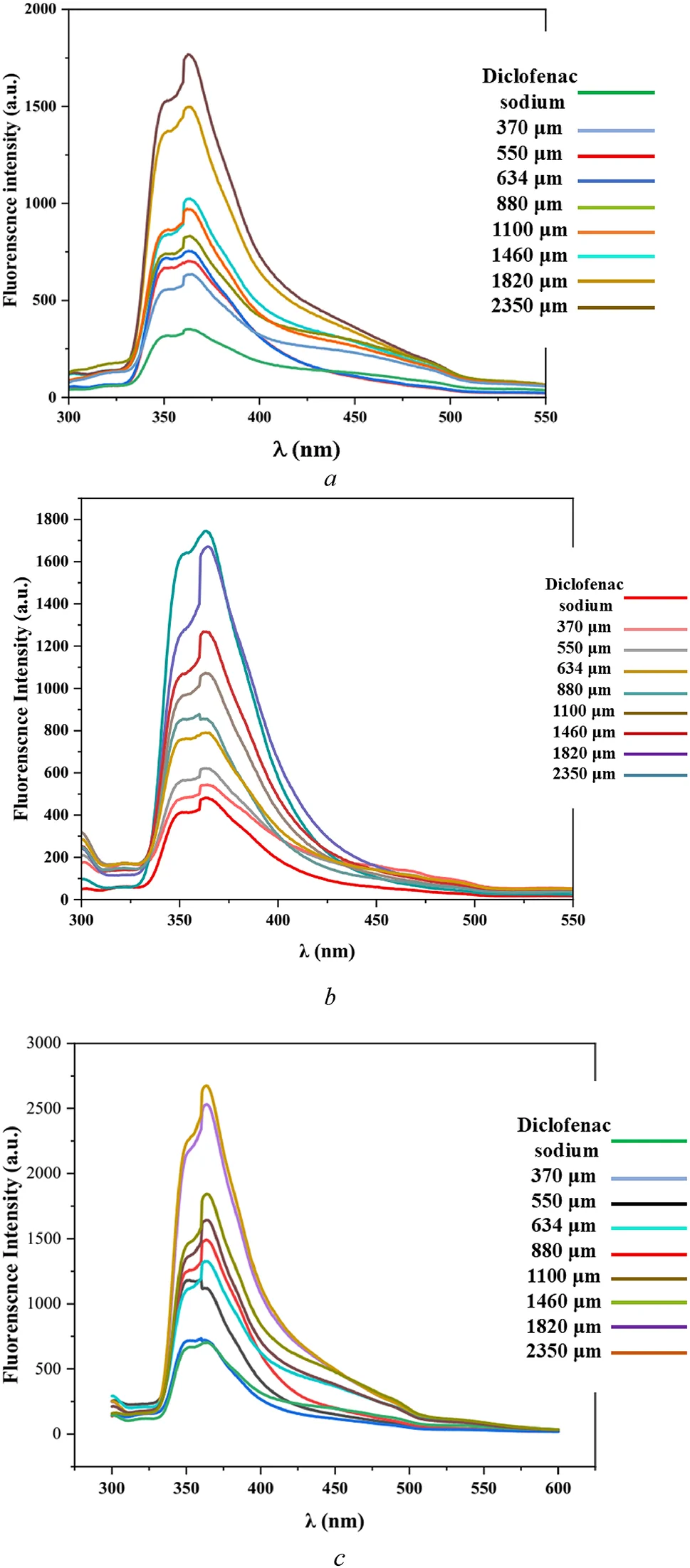
Fluorescence spectra of DFS in presence of different SAILs (with concentration of (370, 550, 634, 880, 1100, 1460, 1820, and 2350) 10^− 6^mol kg^− 1^ (µm)) at 298.15 K; (**a**) Diclofenac sodium in the presence of the [2-HEA][Ole]; (**b**) Diclofenac sodium in the presence of the [BHEA][Ole]; (**c**) Diclofenac sodium in the presence of the [THEA][Ole].

Notably, the solubility of DFS exhibited a monotonic enhancement with increasing temperature and SAIL concentration. These observations underscore a marked elevation in DFS solubility within aqueous SAIL media relative to pure water^25,26^, a phenomenon ascribable to specific intermolecular interactions between DFS moieties and SAIL constituents, as illustrated in Fig. 3.

The hierarchical order of DFS solubility across the investigated SAILs was [2-HEA][Ole] > [BHEA][Ole] > [THEA][Ole].

DFS’s increased solubility in SAILs can be attributed to a thorough interaction of intermolecular forces. The solubilization of hydrophobic pharmaceuticals in a given solvent is fundamentally governed by the magnitude and character of these interactions. In the DFS–SAIL systems under investigation, several principal non-covalent forces predominate as contributors to this phenomenon. The most important of these is hydrogen bonding, in which donor-acceptor relationships are created by the hydroxyl (-OH) and amino (-NH) moieties present in both DFS and the SAILs. These hydrogen-bonded networks increase the accommodation of DFS in the SAIL milieu by encouraging the formation of solute–solvent adducts. Complementarily, van der Waals dispersions, encompassing dipole–dipole couplings, reinforces the solvation dynamics. Instantaneous electron density perturbations are the source of these dispersive interactions, which produce transient dipoles that promote attractive intermolecular cohesion. Additionally, the ionic constituents of SAILs engender ion–dipole attractions with the electronegative domains of DFS, thereby amplifying the electrostatic stabilization of the solvated species. Collectively, these synergistic intermolecular forces culminate in a pronounced augmentation of DFS solubility relative to neat aqueous media. In pure water, DFS solvation is predominantly mediated by hydrogen bonding and dipole–dipole interactions with solvent dipoles; however, the incorporation of SAILs introduces ancillary, more potent electrostatic contributions principally ion–dipole interactions that engender a thermodynamically superior solvation milieu for the solute^27–35^.

To evaluate the effectiveness of the studied SAILs as solubilizing agents, the solubility enhancement of DFS was compared with literature‑reported systems. Conventional surfactants such as SDS have been reported to enhance the aqueous solubility of diclofenac sodium by approximately (3 to 6)‑fold, while nonionic surfactants such as Tween‑80 typically achieve (5 to10)‑fold enhancement. Cholinium‑based ionic liquids have shown improvements in the range of (4 to 12)‑fold, depending on their structural features. In the present study, the hydroxyethylamine‑based SAILs produced a substantial increase in the solubility of diclofenac sodium, particularly in the presence of [THEA][Ole]. The observed enhancement falls within or exceeds the ranges reported for conventional surfactants and cholinium‑based ionic liquids, demonstrating that these bio‑based SAILs are competitive and potentially superior solubilizing media for poorly water‑soluble pharmaceutical compounds^16,20,22,23,108,24^ .

### Correlation procedure

Thermodynamic modeling is crucial for quantifying drug solubility and establishing systematic solvent selection criteria in pharmaceutical formulation engineering.^36–38^. Precise determination of solubility in heterogeneous solvent environments is accomplished through the solution of equilibrium thermodynamic relations. Contemporary theoretical models for solubility prognostication routinely integrate local composition theories, which meticulously accommodate the subtleties of molecular interactions. These frameworks, in particular, elucidate the short-range ordering and non-random molecular alignments that emerge from disparities in molecular volumes and intermolecular potentials within the solution phase^39,40^. These thermodynamic equations incorporate the excess molar Gibbs energy (*G*^ex^) to encapsulate the deviations from ideality. The solubility of the solute in a given solvent mixture at designated temperatures is ascertained by equating the chemical potential (or activity) of the solute in the saturated liquid phase to that in the pure solid phase. This phase equilibrium stipulation is formalized within a solid–liquid equilibrium (SLE) paradigm, which furnishes the following relation:

2 $$Inx_{1} = - In\gamma _{1} + \frac{{\Delta _{{fus}} H}}{R}\left( {\frac{1}{{T_{{m1}} }} - \frac{1}{T}} \right) - \frac{1}{{RT}}\int_{{T_{{m1}} }}^{T} {T\Delta C_{{P1}} dt + \frac{1}{R}\int_{{T_{{m1}} }}^{T} {T\frac{{\Delta C_{{P1}} }}{T}dt} }$$

Within this relation, the symbols denote the following: *R* is the universal gas constant, *T* is the absolute temperature under investigation, and *T*_m1_ is the normal melting point of the solute. The parameters $\Delta _{{fus}} H,\,\gamma _{1}$, and ∆*C*_p1_ correspond to the standard enthalpy of fusion, the activity coefficient of the solute in the saturated solution, and the molar heat capacity increment between the liquid and solid phases of diclofenac sodium (DFS), respectively. Through the application of reasonable approximations, the aforementioned equation simplifies to the following expression^41^:

3 $$In\,x_{1} = \frac{{\Delta _{{fus}} H}}{R}\left( {\frac{1}{{T_{m} }} - \frac{1}{T}} \right) - In\,\gamma _{1}$$

To obtain the experimental solubility data of DFS, it is necessary to determine the melting temperature (*T*_m_), activity coefficient (γ_1_), and enthalpy of fusion ($\Delta _{{fus}} H$). In this study, the correlation of these parameters has facilitated the analysis. The molar excess Gibbs energy (*G*^ex^) is formulated as the superposition of two distinct contributions, thereby enabling the extension of the electrolyte non-random two-liquid (e-NRTL) and Wilson models to multicomponent systems encompassing electrolytes in aqueous media^26,41^:

4 $$\frac{{G^{{ex*}} }}{{RT}} = \frac{{G^{{ex*,LR}} }}{{RT}} + \frac{{F^{{ex*,SR}} }}{{RT}}$$

The superscript ∗ signifies the asymmetric reference state convention, wherein SR denotes short-range interactions and LR designates long-range electrostatic contributions. The extended Pitzer–Debye–Hückel model for the molar excess Gibbs energy (*G*^ex*^), as formulated by Pitzer (1991)^42^, provides an efficacious framework for delineating the long-range interactions prevalent in electrolyte solutions. Concurrently, the short-range interactions are modeled via the electrolyte non-random two-liquid (*e*-NRTL) and Wilson local composition theories.

#### Wilson model

In the Wilson local composition model, the activity coefficients principally modulated by compositional fractions and temperature are formulated according to the ensuing equations^30^:

5 $$In\,\gamma _{i} = 1 - In\left( {\sum\limits_{{j = 1}}^{n} {\left( {\Lambda _{{ij}} x_{j} } \right)} - \sum\limits_{{k = 1}}^{n} {\left( {\frac{{\Lambda _{{ki}} x_{k} }}{{\sum\limits_{{j = 1}}^{n} {\left( {\Lambda _{{kj}} x_{j} } \right)} }}} \right)} } \right)$$

In the aforementioned equations, the parameter *λ* signifies the binary interaction coefficient, which is contingent upon the characteristic interaction energy $\Lambda _{{ij}}$and the molar volumes of the solute and solvent (ν). The computation of this parameter is affected through Eq. (6)^26^:

6 $$\Lambda _{{ij}} = \frac{{{\upsilon }_{j} }}{{{\upsilon }_{i} }}\exp \left( { - \frac{{\lambda _{{ij}} - \lambda _{{ii}} }}{{RT}}} \right)$$

#### Electrolyte-NRTL model

The application of the local composition concept in the context of the electrolyte Nonrandom Two-Liquid (*e*-NRTL) equation has been extensively discussed in previous studies^27–33,35,41^. The activity coefficients for individual species are computed through the additive superposition of the Pitzer–Debye–Hückel electrostatic contribution and the local composition NRTL term. This hybrid formulation facilitates the determination of ($\gamma _{1}$) via Eqs. (7) and (8)^43,44^:

7 $$In\left( {\gamma _{i}^{*} } \right) = In\,\left( {\gamma _{i}^{{*PDH}} } \right) + In\,\left( {\gamma _{i}^{*} NRTL} \right)$$

8 $$In\gamma _{i} = \frac{{\sum\limits_{{j = 1}}^{m} {\tau _{{ij}} G_{{ij}} x_{j} } }}{{\sum\limits_{{i = 1}}^{m} {G_{{li}} x_{l} } }} + \sum\limits_{{j = 1}}^{m} {\frac{{x_{j} G_{{ij}} }}{{\sum\limits_{{l = i}}^{m} {G_{{lj}} x_{l} } }}}$$

9 $$\tau _{{ij}} = \frac{{\Delta g_{{ij}} }}{{RT}} = \frac{{g_{{ij}} - g_{{ii}} }}{{RT}},G_{{ij}} = \exp ( - \alpha \frac{{\Delta g_{{ij}} }}{{RT}})$$

In the e-NRTL formulation, the parameter $G_{{ij}}$ were identified as as$G_{{ij}} = \exp \left( { - \alpha _{{ij}} \tau _{{ij}} } \right)$and $\tau _{{ii}} = \tau _{{ij}} = 0$ respectively, with $\alpha _{{ij}} = \alpha _{{ji}}$ denoting the non-randomness factor. Additionally, Eq. (9) was employed to compute the binary interaction parameter ($\tau _{{ij}}$). The term *g*_ij_ represents the characteristic energy parameter governing the pairwise i–j interactions. Ultimately, the model interaction parameters were regressed by minimizing the objective function delineated in Eq. (10).

10 $$OF = \sum\limits_{{i = 1}}^{n} {\left( {In\gamma _{i}^{{\exp }} - In\gamma _{i}^{{cal}} } \right)} ^{2}$$

The activity coefficients associated with these electrostatic and local compositional states are designated as $In\gamma _{i}^{{cal}}$ and $In\gamma _{i}^{{\exp }}$ ., respectively. To quantify the discrepancies between the predicted $\left( {x_{i}^{{cal}} } \right)$ and observed $\left( {x_{i}^{{\exp }} } \right)$ solubilities, the average relative deviation (*ARD*) percentage is invoked. This statistical measure is evaluated through Eq. (11), which is germane to the aforementioned thermodynamic frameworks. In this expression, *N* signifies the aggregate number of experimental data points:

11 $$ARD = 100\left( {\frac{{\sum\limits_{{i = 1}}^{N} {\left| {x_{i}^{{\exp }} - x_{i}^{{cal}} } \right|} }}{N}} \right)$$

The experimental solubility data were regressed against the Wilson and electrolyte non-random two-liquid (e-NRTL) models, with the resultant interaction parameters and predictive solubilities compiled in Table 2. Moreover, Table 3 enumerates the average relative deviation (ARD) percentages, which exhibit consistently low values, thereby substantiating the robust predictive performance of these models in delineating the mole fraction solubility of diclofenac sodium (DFS) within the examined systems^45–47^.

**Table 3:** The calculated average relative deviation percent (*ARD%*) for the solubility of the DFS in the aqueous SAILs solutions at the temperature ranges *T*/K = (298.15 to 313.15) and pressure (*p* = 866 hPa) from different models.

ARD%		
*T* / K	*e*-NRTL	Wilson
DFS (1) + Water (2) + [2-HEA][Ole] (3)		
298.15	4.71	0.30
303.15	4.88	0.11
308.15	3.69	0.36
313.15	2.60	0.01
Average	3.97	0.19
DFS (1) + Water (2) + [BHEA][Ole] (3)		
298.15	2.25	0.11
303.15	3.65	0.23
308.15	4.78	0.46
313.15	4.36	0.05
Average	3.76	0.85
DFS (1) + Water (2) + [THEA][Ole] (3)		
298.15	5.08	0.36
303.15	4.52	0.71
308.15	3.74	0.06
313.15	4.01	0.08
Average	4.33	0.30

The regression analyses demonstrate a robust concordance between the thermodynamic models and the empirical solubility data, underscoring their superior predictive fidelity. The correlative performance of the thermodynamic models was evaluated using the average relative deviation (ARD%). The ARD% values were calculated according to Eq. (11) using all experimental solubility data points. Consequently, the overall ARD values reported in Table 3 represent pooled deviations over the entire dataset rather than the arithmetic mean of the individual system ARDs. Based on this pooled calculation, the Wilson and *e*‑NRTL models yielded overall ARD values of 0.20% and 5.34%, respectively. Therefore, the individual system ARD values do not necessarily average to the reported overall ARD.

### The apparent dissolution thermodynamic properties

The thermodynamic properties of the dissolution process were determined by evaluating apparent thermodynamic functions using the Gibbs and van’t Hoff equations. These calculations were performed at the mean harmonic temperature ($T_{{hm}} = \frac{N}{{\sum\limits_{{i = 1}}^{N} {\frac{1}{{T_{i} }}} }}$, *T*_hm_ = 305.55 K), which was obtained from the temperature range of (298.15 to 313.15) K^48–51^. The standard molar enthalpy of dissolution ($\Delta H_{{so\ln }}^{ \circ }$) for DFS was determined using Eq. (12):

12 $$\Delta H_{{so\ln }}^{ \circ } = - R\left( {\frac{{\partial Inx_{1} }}{{\partial \left( {{\raise0.5ex\hbox{$\scriptstyle 1$} \kern-0.1em/\kern-0.15em \lower0.25ex\hbox{$\scriptstyle T$}}} \right)}}} \right)p$$

In the aforementioned equation, x_1_ designates the mole fraction solubility of diclofenac sodium (DFS), *R* denotes the universal gas constant (8.314 J K^-1^ mol^-1^), and *T* represents the absolute temperature. The $$\Delta H_{{so\ln }}^{ \circ }$$ was additionally ascertained via linear regression of the van’t Hoff plot, wherein ln *x*_1_ is graphed against $${\raise0.5ex\hbox{$\scriptstyle 1$} \kern-0.1em/\kern-0.15em \lower0.25ex\hbox{$\scriptstyle T$}} - {\raise0.5ex\hbox{$\scriptstyle 1$} \kern-0.1em/\kern-0.15em \lower0.25ex\hbox{$\scriptstyle {T_{{hm}} }$}}$$, with the slope yielding $$\Delta H_{{so\ln }}^{ \circ }$$^41^

13 $$\Delta H_{{so\ln }}^{ \circ } = - R\left( {\frac{{\partial Inx_{1} }}{{\partial \left( {{\raise0.5ex\hbox{$\scriptstyle 1$} \kern-0.1em/\kern-0.15em \lower0.25ex\hbox{$\scriptstyle T$}} - {\raise0.5ex\hbox{$\scriptstyle 1$} \kern-0.1em/\kern-0.15em \lower0.25ex\hbox{$\scriptstyle {T_{{hm}} }$}}} \right)}}} \right)p$$

14 $$\Delta G_{{so\ln }}^{ \circ } = - RT_{{hm}} \times \mathrm{int} ercept$$

By leveraging the slopes and intercepts obtained from Eqs. (13) and (14), the ΔH_soln_°, ΔG_soln_°, and *T*$\Delta H_{{so\ln }}^{ \circ }$, changes associated with dissolution were determined. Furthermore, the standard molar entropy of dissolution ($T\Delta S_{{so\ln }}^{ \circ }$) was calculated using the following relation:

15 $$\Delta S_{{so\ln }}^{ \circ } = \frac{{\Delta H_{{so\ln }}^{ \circ } - \Delta G_{{so\ln }}^{ \circ } }}{{T_{{hm}} }}$$

Eventually, in the DFS dissolving process, Eqs. (16) and (17) have been applied to compare the relative contributions to the standard molar Gibbs free energy by enthalpy and entropy, as evidenced by the $\xi _{H}$ and $\xi _{{TS}}$ values^26,41^:

16 $$\% \xi _{H} = \frac{{\left| {\Delta H_{{so\ln }}^{ \circ } } \right|}}{{\left| {\Delta H_{{so\ln }}^{ \circ } } \right| + \left| {T\Delta S_{{so\ln }}^{ \circ } } \right|}} \times 100$$

17 $$\% \xi _{{TS}} = \frac{{\left| {T\Delta S_{{so\ln }}^{ \circ } } \right|}}{{\left| {\Delta H_{{so\ln }}^{ \circ } } \right| + \left| {T\Delta S_{{so\ln }}^{ \circ } } \right|}} \times 100$$

To elucidate the thermodynamic attributes of the dissolution process, van’t Hoff plots of () versus for DFS in aqueous SAILs solutions were constructed. The derived thermodynamic parameters of ($\Delta G_{{so\ln }}^{ \circ }$), ($\Delta H_{{so\ln }}^{ \circ }$), and ($T_{m} \Delta S_{{so\ln }}^{ \circ }$) are tabulated in Table 4.

**Table 4:** Thermodynamic functions for dissolution process at different weight fractions of PILs (w3) at mean harmonic temperature (Thm = 305.55 K).

w_3_	$${\raise0.5ex\hbox{$\scriptstyle {\Delta H_{{so\ln }}^{ \circ } }$} \kern-0.1em/\kern-0.15em \lower0.25ex\hbox{$\scriptstyle {{\mathrm{kJ}}\,{\mathrm{mol}}^{{ - {\mathrm{1}}}} }$}}$$	$$T_{{hm}} \Delta S_{{so\ln }}^{^\circ }$$/ kJ∙mol^− 1^	$$\Delta G_{{so\ln }}^{^\circ }$$/ kJ∙mol^− 1^	$$\:{\xi\:}_{H}$$	$$\:{\xi\:}_{TS}$$
DFS (1) + Water (2) + [2-HEA][Ole] (3)					
0.0000	7.87	-15.93	23.80	33.08	66.92
0.1000	24.09	5.37	18.72	81.76	18.24
0.3000	11.28	-6.96	18.24	61.86	29.46
0.5000	17.95	0.86	17.09	95.44	4.56
0.7000	52.86	36.87	15.98	58.91	41.09
0.9000	35.65	22.42	13.23	61.39	38.60
1.0000	4.54	-5.18	11.23	46.68	12.70
DFS (1) + Water (2) + [BHEA][Ole] (3)					
0.0000	7.87	-15.93	23.80	33.08	66.92
0.1000	39.23	20.14	19.09	66.08	33.92
0.3000	38.43	19.68	18.75	66.13	34.66
0.5000	50.05	32.23	17.82	60.83	39.17
0.7000	48.50	31.93	16.58	60.30	39.69
0.9000	30.48	17.07	13.41	64.10	35.90
1.0000	23.15	10.45	12.70	68.89	25.54
DFS (1) + Water (2) + [THEA][Ole] (3)					
0.0000	7.87	-15.93	23.80	33.08	66.92
0.1000	37.52	18.21	19.30	67.32	32.68
0.3000	37.92	19.12	18.80	66.48	31.93
0.5000	50.44	32.36	18.09	60.92	39.08
0.7000	68.82	52.28	16.54	56.83	43.17
0.9000	85.28	69.21	14.68	55.20	44.80
1.0000	44.52	30.03	14.19	59.72	26.04

It is noteworthy that, across all examined systems, the standard molar Gibbs free energy (ΔG°) and dissolution enthalpy (ΔH°) associated with the solubilization of DFS in aqueous surface-active ionic liquid (SAIL) media exhibited uniformly positive values, thereby signifying that the dissolution processes are invariably endothermic.

The standard molar Gibbs free energy changes ($\Delta G_{{so\ln }}^{ \circ }$) diminish with escalating weight fraction of SAIL, thereby signifying an augmentation in the solubility of DFS within these solvent systems as ($\Delta G_{{so\ln }}^{ \circ }$) assumes more negative values. In contrast, the standard dissolution entropy ($\Delta S_{{so\ln }}^{ \circ }$) exhibits uniformly positive magnitudes across all solutions interrogated in this study, with the $T\Delta S_{{so\ln }}^{ \circ }$ terms being inferior in magnitude to their corresponding $H_{{so\ln }}^{ \circ }$ counterparts. Table 5 tabulates the computed ($\xi _{H}$) and ($\xi _{{TS}}$) values. These observations substantiate that the enthalpic contribution predominates in governing the standard molar Gibbs free energy of the DFS dissolution process. The thermodynamic analysis indicates that both enthalpic and entropic contributions participate in the dissolution process. However, for most SAIL compositions, the enthalpic contribution ($\:{\xi\:}_{H}$) exceeds the entropic contribution ($\:{\xi\:}_{TS}$), demonstrating that the solubilization process is predominantly enthalpy-driven over the investigated composition range^33,52,53^. The thermodynamic parameters obtained from the van’t Hoff analysis provide insight into the mechanism of DFS dissolution in aqueous SAIL solutions. The positive values of $\:{\Delta\:}{H}^{\circ\:}$indicate that the dissolution process is endothermic, suggesting that energy is required to overcome the crystal lattice energy of DFS and to disrupt the hydrogen-bonded structure of water. This process is accompanied by favorable entropy changes ($\:{\Delta\:}{S}^{\circ\:}>0$), reflecting an increase in system disorder. This entropy gain is attributed to the disruption of structured water molecules surrounding the hydrophobic regions of both DFS and the oleate-based SAILs. Furthermore, the amphiphilic nature of the SAILs promotes the formation of micelle-like aggregates, providing hydrophobic microenvironments that enhance the solubilization of the drug. The incorporation of DFS into these domains releases ordered water molecules into the bulk phase, further contributing to the entropy of the system. However, a comparison of the relative contributions shows that $\:\mid\:{\xi\:}_{H}\mid\:\:>\:\mid\:\left.{\xi\:}_{TS}\right|\:$. Consequently, despite the favorable entropic contribution, the dissolution process is predominantly enthalpy-driven, indicating that the energetic interactions between the drug and the SAIL-water environment are the primary factors governing the solubility behavior.

### Modeling results

The findings from the thermodynamic modeling of diclofenac sodium (DFS) solubility in binary aqueous surface-active ionic liquid (SAIL) mixtures, encompassing an array of physicochemical parameters, are compiled in Tables 5 and 6. The interaction parameters regressed through the electrolyte non-random two-liquid (e-NRTL) model for DFS solubility in aqueous SAIL media are delineated in Table 5.

**Table 5:** The parameters of the *e*-NRTL model were applied to analyze the behavior of DFS in aqueous SAIL solutions.

*T* (K)	*Δ*g_DW_	*Δ*g_WD_	*Δ*g_Cad_	*Δ*g_dCa_	*Δ*g_CaW_	*Δ*g_WCa_
DFS (1) + water (2) + [2-HEA] [Ole] (3)						
298.15	2.649 × 10^3^	-0.848	-1.529 × 10^5^	3.779 × 10^5^	2.716 × 10^4^	5.594 × 10^5^
303.15	1.27 × 10^3^	371.155	-4.073 × 10^3^	917.979	1.082 × 10^5^	1.966 × 10^5^
308.15	2.681 × 10^3^	0.349	-2.825 × 10^3^	5.353 × 10^3^	1.284 × 10^4^	-7.435 × 10^3^
313.15	2.743 × 10^3^	858.359	-2.945 × 10^3^	2.889 × 10^4^	3.954 × 10^3^	6.568 × 10^4^
DFS (1) + water (2) + [BHEA][Ole] (3)						
298.15	7.759 × 10^3^	-0.425	1.243 × 10^4^	1.281 × 10^3^	1.171 × 10^3^	506.35
303.15	1.049 × 10^4^	-1.75 × 10^3^	2.771 × 10^3^	411.554	1.082 × 10^5^	1.966 × 10^5^
308.15	9.646 × 10^3^	-1.59 × 10^3^	5.787 × 10^3^	2.552 × 10^6^	1.413 × 10^3^	-1.14 × 10^4^
313.15	0.097	1.029 × 10^3^	884.988	2.059 × 10^5^	4.766 × 10^3^	-7.15 × 10^5^
DFS (1) + water (2) + [THEA][Ole] (3)						
298.15	9.157 × 10^3^	-832.824	9.343 × 10^4^	-3.378 × 10^4^	4.59 × 10^3^	-3.212 × 10^4^
303.15	9.426 × 10^3^	-1.496 × 10^3^	1.094 × 10^4^	-27.56	1.082 × 10^5^	1.966 × 10^5^
308.15	9.771 × 10^3^	-1.784 × 10^3^	5.945 × 10^3^	2.552 × 10^6^	1.416 × 10^3^	-1.14 × 10^4^
313.15	0.087	871.028	-728.481	2.059 × 10^5^	3.811 × 10^3^	-7.15 × 10^5^

*W* = water, *D* = drug (DFS), *Ca* = cation((2-hydroxyethyl) amine, bis(2-hydroxyethyl)amine, and tris(2-hydroxyethyl)amine) and anion (Oleic acid).

The interaction parameters regressed via the Wilson model for diclofenac sodium (DFS) solubility in aqueous surface-active ionic liquid (SAIL) solutions are enumerated in Table 6.

**Table 6:** The parameters of the Wilson model were applied to analyze the behavior of DFS in aqueous SAIL solutions.

*T* (K)	*Λ*_DW_	*Λ*_WD_	*Λ*_Cad_	*Λ*_dCa_	*Λ*_CaW_	*Λ*_WCa_
DFS (1) + water (2) + [2-HEA] [Ole] (3)						
298.15	3.429	5.819 × 10^− 6^	3.003 × 10^3^	-0.013	720.903	-1.215 × 10^− 4^
303.15	3.265	-3.138 × 10^− 7^	1.633 × 10^3^	-6.096 × 10^− 3^	189.06	1.578
308.15	3.096	2.004 × 10^− 7^	2.791 × 10^− 3^	2.08 × 10^− 3^	1.025 × 10^− 5^	-1.073
313.15	2.936	2.731 × 10^− 6^	6.984 × 10^− 4^	2.213 × 10^− 3^	-2.967 × 10^− 3^	-1.201
DFS (1) + water (2) + [BHEA][Ole] (3)						
298.15	3.418	4.261 × 10^− 5^	1.086 × 10^3^	-3.903 × 10^− 3^	1.093 × 10^3^	-1.215 × 10^− 4^
303.15	3.248	5.915 × 10^− 5^	1.633 × 10^3^	-6.096 × 10^− 3^	189.081	1.578
308.15	3.089	-2.695 × 10^− 6^	2.791 × 10^− 3^	2.08 × 10^− 3^	1.025 × 10^− 5^	-1.073
313.15	2.931	-3.675 × 10^− 7^	7.346 × 10^− 4^	2.213 × 10^− 3^	-3.74 × 10^− 3^	-1.481
DFS (1) + water (2) + [THEA][Ole] (3)						
298.15	3.422	9.296 × 10^− 6^	1.801 × 10^3^	-2.03 × 10^− 3^	1.02 × 10^3^	-1.215 × 10^− 4^
303.15	3.26	-5.777 × 10^− 7^	1.617 × 10^3^	-6.096 × 10^− 3^	190.924	1.587
308.15	3.095	1.279 × 10^− 7^	2.884 × 10^− 3^	2.08 × 10^− 3^	1.025 × 10^− 5^	-1.464
313.15	2.936	-1.931 × 10^− 7^	4.831 × 10^− 4^	2.213 × 10^− 3^	-4.22 × 10^− 3^	-1.541

### Intrinsic fluorescence spectroscopy analysis

Fluorescence spectroscopy is a sensitive and effective technique for investigating molecular interactions, based on the radiative deactivation of electronically excited states. Quenching agents attenuate fluorescence emission intensity, providing critical insights into intermolecular associations and the formation of fluorophore–quencher complexes^54,55^. In this study, steady-state fluorescence was employed to characterize the binding affinity between DFS, serving as the fluorophore, and the hydroxyethyl-derived SAILs acting as quenchers. The emission spectra of DFS at 298.15 K, recorded in the absence and presence of increasing SAIL concentrations, are presented in Fig. 3.

The emission maximum of DFS was observed at approximately 360 nm upon excitation at λex = 260 nm, with spectra recorded over the 265–625 nm emission range. As shown in Fig. 3, the fluorescence intensity of DFS gradually decreased with increasing concentrations of each surface‑active ionic liquid (SAIL), indicating a concentration‑dependent quenching effect. This behavior suggests effective interaction between DFS molecules and the SAIL aggregates in aqueous solution.

The quenching may be attributed to the incorporation of DFS into the micellar aggregates formed by the SAILs above their critical micelle concentration (CMC). Such interactions can facilitate non‑radiative relaxation processes or promote collisional encounters between the excited fluorophore and the quencher species within the organized micellar environment. Notably, the emission maximum remained essentially unchanged at 360 nm, indicating that the local microenvironment polarity around the fluorophore does not undergo significant alteration. Similar fluorescence quenching behavior has been reported for hydrophobic anionic drugs interacting with amphiphilic surfactant systems^22,56–60^. The relative quenching efficiency of the investigated SAILs follows the order: [THEA][Ole] > [BHEA][Ole] > [2‑HEA][Ole].

This quenching profile demonstrates a pronounced affinity between DFS and the SAILs, particularly [THEA][Ole]. Fluorescence quenching, broadly defined, is a reduction in a fluorophore’s emission intensity resulting from various molecular interactions. These can include conformational changes, Förster resonance energy transfer (FRET), excited-state proton transfer, collisional deactivation, and ground-state complexation. Distinguishing between dynamic and static quenching mechanisms is crucial for elucidating the nature of these interactions. Dynamic quenching occurs when the excited-state fluorophore (DFS*) and the quencher (SAIL) collide during the excited state lifetime. These collisional encounters dissipate energy, reducing the radiative decay pathway and thus the fluorescence quantum yield (Φf) and emission. In contrast, static quenching results from the formation of a non-emissive ground-state complex between the fluorophore and quencher before excitation, rendering the complex photo physically inert^61–64^. The differentiation between these quenching modalities is typically achieved through the application of the Stern–Volmer equation^65^:

18 $$\frac{{F_{0} }}{F} = 1 + K_{{SV}} C_{Q} = 1 + K_{q} \tau _{0} C_{Q}$$

19 $$\frac{{F_{0} }}{F} = e^{{K_{{SV}} C_{Q} }}$$

20 $$K_{A} = \frac{{K_{{SV}} }}{{K_{q} }}$$

In the Stern–Volmer equation, *F*_0_ and *F* denote the fluorescence intensities of the fluorophore in the absence and presence of the quenching agent, respectively; *C*_Q_ represents the molar concentration of the quencher; and *K*_SV_ signifies the Stern–Volmer quenching constant, expressible as $K_{{SV}} = K_{q} \tau _{0}$, wherein $K_{q}$ is the bimolecular quenching rate constant and $\tau _{0}$ is the average fluorescence lifetime of the drug in the absence of quencher. Equations (18) and (19) constitute the classical and modified Stern–Volmer relations, respectively, which facilitate the mechanistic interrogation of fluorescence quenching data through linear regression of *F*_*0*_*/F* versus *C*_Q_^66^.

The association constant $\:{K}_{A}$, as defined in Eq. (20), represents the equilibrium constant describing the interaction between DFS and the SAIL molecules. Higher $\:{K}_{A}$values indicate stronger binding affinity and greater stability of the resulting DFS–SAIL complex. The magnitude of $\:{K}_{A}$, together with the Stern–Volmer parameters, provides insight into the interaction strength and supports the proposed quenching mechanism in the DFS–SAIL system^67–70^.

**Fig. 4:**
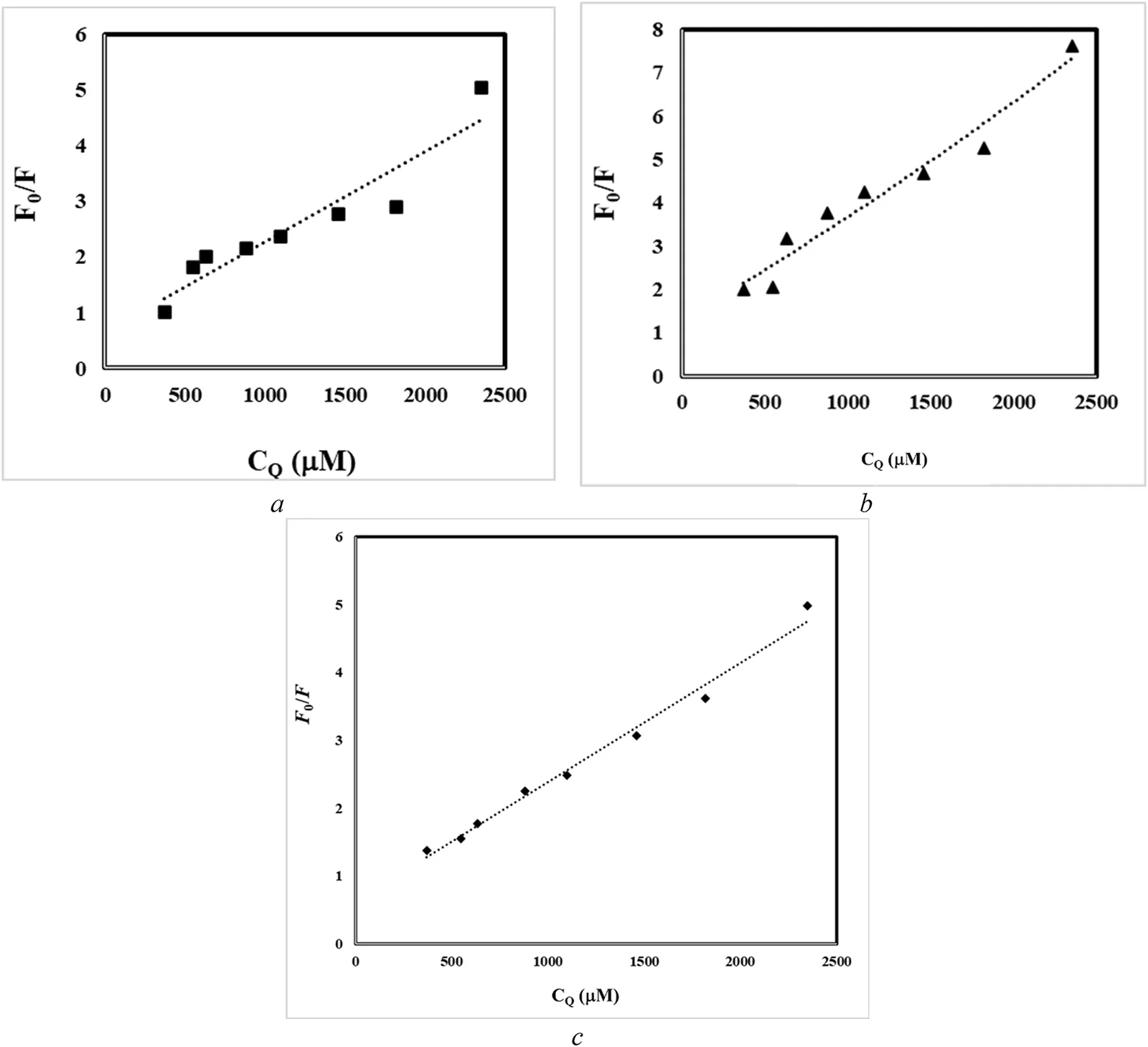
The Stern-Volmer plot of the Diclofenac sodium-SAILs (with concentration of (370, 550, 634, 880, 1100, 1460, 1820, and 2350) 10^− 6^mol kg^− 1^ (µm)) at 298.15 K; (**a**) Diclofenac sodium in the presence of the [2-HEA][Ole]; (**b**) Diclofenac sodium in the presence of the [BHEA][Ole]; (**c**) Diclofenac sodium in the presence of the [THEA][Ole].

The linearity of the Stern–Volmer plot, as delineated by Eq. (18), is indicative of a singular binding locus proximate to the fluorophore accessible to the quencher, consistent with either static or dynamic quenching mechanisms. In contradistinction, an upward concavity in the plot signifies the concurrence of multiple quenching pathways or the availability of ancillary binding sites. For the quantitative interrogation of such nonlinear Stern–Volmer profiles predominantly observed at elevated quencher concentrations Eq. (19) is employed^71–76^. The Stern-Volmer plots for drug-surface active ionic liquid (SAIL) complexes are presented in Fig. 4.

The intrinsic fluorescence of DFS exhibited a systematic decrease in emission intensity upon incremental addition of the synthesized SAILs, confirming a concentration‑dependent quenching interaction between DFS and the ionic liquid species. This progressive reduction in fluorescence intensity formed the basis for the application of the Stern–Volmer formalism, from which the quenching constants and related interaction parameters were determined. In a few cases, a slight increase in fluorescence intensity was observed at very low SAIL concentrations. This minor enhancement is not associated with quenching and is likely attributable to microenvironmental effects around the fluorophore, such as slight reductions in local solvent polarity or partial shielding from non‑radiative relaxation pathways. These initial enhancement regions were therefore excluded from the Stern–Volmer analysis. Consequently, all quantitative fluorescence parameters were calculated only within the concentration range where genuine quenching behavior was observed^72,77^.

The K_q_ and K_SV_ values of DFS in the examined SAIL solutions are listed in Table 7. In fluorescence systems, the maximum diffusion-controlled collisional quenching constant is typically about 2 × 10^10^ M^− 1^∙s. Since the calculated K_q_ values in the present study are significantly higher than this limiting value, dynamic quenching alone cannot explain the observed decrease in fluorescence intensity. This finding suggests that static interactions, including complex formation between DFS and the SAIL molecules, make an important contribution to the quenching process^78,79^.

**Table 7:** The quenching constants (K_sv_ and K_q_) and binding site number (*n*) of the interaction between diclofenac sodium and different surface active ionic liquid at 298.15 K.^*a*^.

SAILs	K_SV_(M^− 1^)	K_q_(10^12^ M^−1^s^− 1^)	K_A_(10^4^ M^− 1^)	*n*
[2-HEA][Ole]	1.714	0.857	2.785	1.09
[BHEA][Ole]	2.581	1.291	1.328	0.87
[THEA][Ole]	1.732	0.866	1.059	0.91

^*a*^ The standard uncertainty in temperature measurements is *u*(*T*) = 0.1 K.

^*b*^ The standard uncertainty, *u*_*c*_, associated with the dynamic Stern–Volmer quenching, $K_{{SV}}$, bimolecular quenching constant, $K_{q}$, and static quenching constant, $K_{A}$, are *u*_*c*_ ($K_{{SV}}$) = 0.06 M^− 1^, *u*_*c*_ ($K_{q}$) = 0.15 × 10^− 12^ M^− 1^.s^− 1^, and *u*_*c*_ ($\:{K}_{A}$) = 0.06 × 10^− 4^ M^− 1^, respectively.

In the present study, the Stern–Volmer quenching constant (K_SV_) and the bimolecular quenching rate constant (K_q_) for the investigated DFS–SAIL systems were determined to be on the order of M^− 1^ and 10^12^ M^− 1^∙s, respectively. Notably, the $K_{q}$ values significantly exceed the diffusion controlled limit for dynamic quenching $K_{{q,r}}$≈ 2 × 10^12^ M^− 1^ s^− 1^ reported in aqueous systems^54,80^. This observation indicates that purely collisional dynamic quenching is unlikely and suggests that static interactions, such as ground state complex formation between DFS and the SAIL molecules, contribute significantly to the observed quenching behavior^81^.

**Fig. 5:**
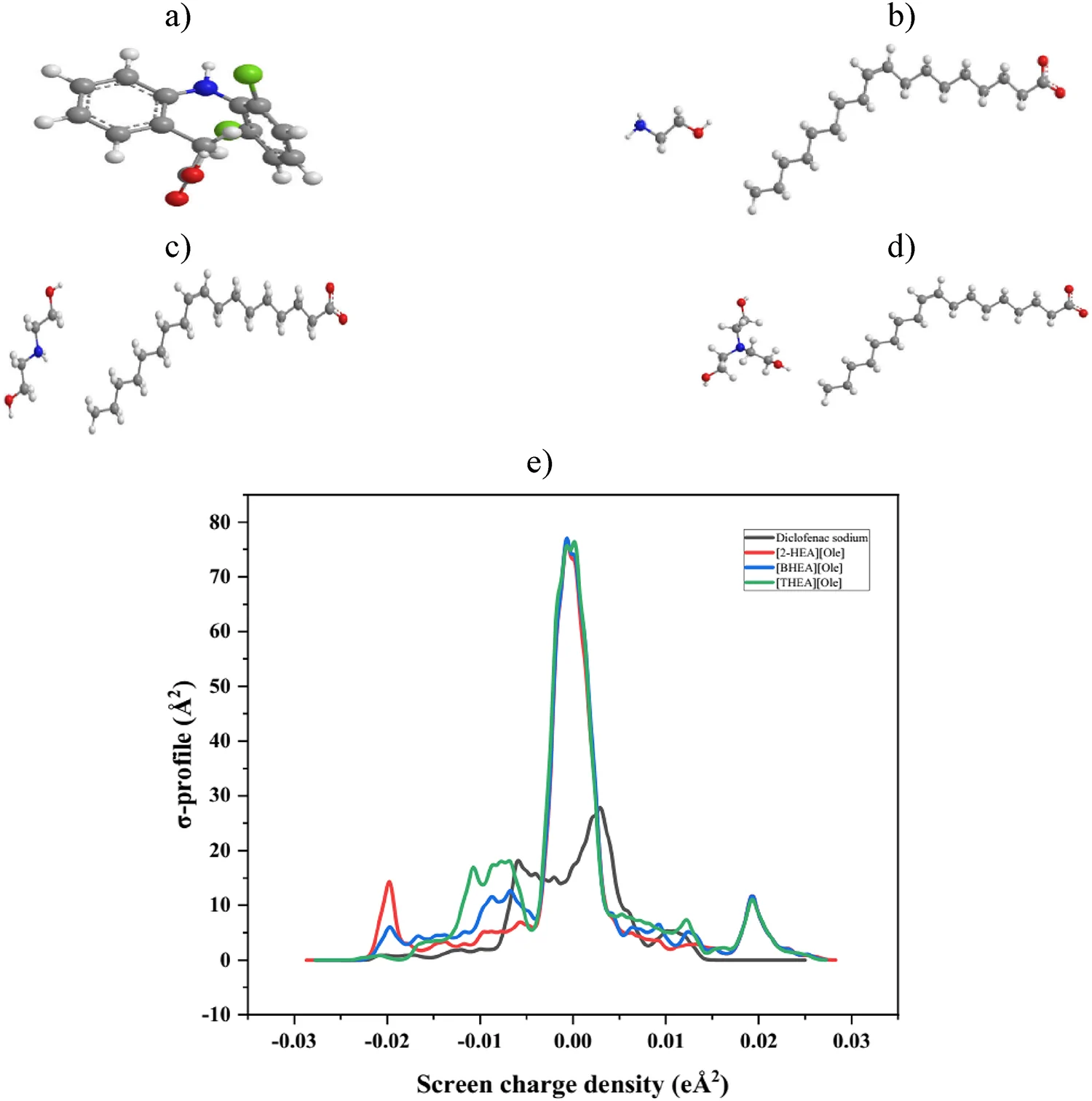
Optimized molecular geometries (computed using BIOVIA Materials Studio DMol³) and *σ*-profiles for (**a**) diclofenac sodium, (**b**) [2-HEA][Ole], (**c**) [BHEA][Ole], and (**d**) [THEA][Ole]; (**e**) overlaid σ-profile distributions obtained from DMol³ and COSMO computations.

The quenching constants ($\:{K}_{SV}$ and $\:{K}_{q}$) listed in Table 7 characterize the intermolecular interactions between DFS and the investigated SAILs. While $\:{K}_{q\:}$is often used to describe collisional frequency, the observed magnitudes in this study significantly exceed the typical values for diffusion-controlled encounters ($\:\:2\times\:{10}^{10}{\mathrm{\:M}}^{-1}{\mathrm{s}}^{-1}$), which strongly suggests a static quenching mechanism. In this context, $\:{K}_{SV}$likely represents the equilibrium propensity for ground-state association, potentially leading to the formation of non-emissive DFS–SAIL adducts. However, it should be noted that in the absence of fluorescence lifetime measurements, a static mechanism is strongly suggested rather than conclusively demonstrated, as lifetime data would be required to definitively distinguish between static and dynamic processes. Furthermore, the binding site stoichiometry (* n*) provides insight into the coordination between the SAIL molecules and the DFS scaffold, where $\:n\approx\:1$ suggests a specific interaction ratio. The binding affinity follows the sequence [THEA][Ole] > [BHEA][Ole] > [2-HEA][Ole], which mirrors the hierarchy observed in the quenching efficiency^82–84^. Furthermore, the Stern–Volmer regression analyses, as evidenced by the linear profiles in Fig. 5, are consistent with a predominantly static quenching mechanism. While the linearity suggests the formation of a single type of complex, the high magnitude of the quenching constants remains the primary indicator of static interaction in this study.

#### Association constants

Assuming that the observed fluorescence quenching of DFS proceeds predominantly through a static mechanism, in which the decrease in fluorescence intensity arises from the formation of a non-emissive ground-state complex with the SAIL quencher, the equilibrium between the free and bound species can be described by the following relation^79^:

21 $$\frac{{\log (F0 - F)}}{F} = \log K_{A} + n.\log C_{Q}$$

The value of *n* denotes the inferred number of binding sites in DFS, specifying the number of quenchers bound to a drug molecule.

The association constants ($\:{K}_{A}$) and binding site stoichiometries (*n*) were derived from the slope and intercept of the linear regression of the $\frac{{\log (F0 - F)}}{F}$ versus $\log C_{Q}$ with the resultant parameters enumerated in Table 7. Across the investigated systems, the *n* value was highest for DFS in aqueous [THEA][Ole] solution, indicating the greatest number of accessible binding sites. Consistently, both K_A_ and *n* followed the order [THEA][Ole] > [BHEA][Ole] > [2‑HEA][Ole], highlighting the stronger interaction of DFS with [THEA][Ole] in terms of binding affinity and complex stability. These observations suggest a direct relationship with the solubilization efficiency of DFS, whereby higher K_A_ and *n* values correspond to enhanced drug solubility in the SAIL media. This interpretation is consistent with previous reports describing the strong solubilizing capability of (2‑hydroxyethyl)amine‑based surface‑active ionic liquids, particularly for poorly water‑soluble pharmaceutical compounds^26,41,44,46,60,85–89^.

## Molecular fingerprints of the SAILs and diclofenac sodium

The theoretical underpinnings of this investigation are predicated upon density functional theory (DFT) computations executed within the DMol³ module, augmented by the conductor-like screening model (COSMO) for solvation effects. Calculations were performed using the Materials Studio suite (BIOVIA, 2023), employing the generalized gradient approximation (GGA) in conjunction with the Vosko–Wilk–Nusair (VWN)–Becke–Perdew (BP) exchange-correlation functional, in accordance with the prescriptive guidelines of the DMol³ developers^90^. Aqueous solvation was explicitly modeled via the COSMO continuum dielectric framework, with water designated as the implicit solvent. The computational protocol entailed a sequential two-stage optimization paradigm: initial geometry relaxation followed by single-point energy refinement, leveraging the GGA VWN-BP functional, the double-numeric density (DND) basis set augmented to 3.5 polarization quality, and incorporation of COSMO-derived solvation free energies. The resultant COSMO outputs, encompassing the surface charge density *σ*-profile and equilibrated molecular geometries of the constituent species, are delineated in Fig. 4. A hallmark of COSMO-derived thermodynamics resides in the *σ*-profile, which functions as a quintessential molecular descriptor encapsulating the probabilistic distribution of screening charge densities across the molecular surface^91,92^. This descriptor furnishes incisive revelations regarding the propensity for localized charge segregation within molecular domains, thereby illuminating the electrostatic topography. Pertinent COSMO variants, such as COSMO with real solvating fluids (COSMO-RS) and segment activity coefficient (COSMO-SAC), harness these *σ*-profiles to prognosticate bulk thermodynamic observables and solvation affinities^93^. Conventionally, *σ*-profiles are extricated from ab initio DFT simulations of molecular electron densities, a process that exacts substantial computational overhead and thereby circumscribes the scalability of such quantum-chemical methodologies. Notwithstanding these exigencies, the *σ*-profile endures as a formidable analytical construct for interrogating intramolecular charge partitioning, affording profound mechanistic perspectives on molecular polarity, electrophilic/nucleophilic susceptibilities, and the tenor of non-covalent intermolecular forces^37,94–96^.

In the investigated SAILs, the *σ*-profiles enable a precise delineation of the charge partitioning between cationic and anionic constituents, a cardinal determinant of their distinctive physicochemical attributes^97^. The COSMO-derived *σ*-profile affords a rigorous quantitative portrayal of the screening charge density distribution across molecular surfaces, thereby furnishing incisive mechanistic perspectives on molecular polarity, electrostatic topography, and non-covalent intermolecular interactions^98^. *σ*  Comparative analysis of the *σ*-profiles for the investigated SAILs and DFS in water reveals significant differences in charge dispersion and localization, which are representative of their fundamental electrical properties. The charge density profiles of the SAILs are typified by broadened peaks spanning approximately (− 0.02 to 0.02) e Å⁻², signifying a pronounced delocalization of electronic charge. This delocalized regime, intrinsic to SAILs, arises from the substantial charge segregation between the ionic moieties, thereby promoting intensified electrostatic couplings and enhanced solubility in polar media. In contrast, the *σ*-profile of DFS exhibits narrower peaks centered at circa − 0.01 e Å⁻², indicative of a more confined electron density. This disparity in charge partitioning is ascribable to the molecular scaffold of DFS, wherein the rigid aromatic framework enforces electronic localization, thereby circumscribing charge delocalization^96^.

In contrast, the incorporation of flexible alkyl moieties and hydroxyl functionalities within theSAILs engenders a more extensive delocalization of electron density over the molecular surface. Comparative analysis of the relative peak intensities in the *σ*-profiles furnishes additional granularity regarding the disparities in electron density partitioning among the scrutinized species.   The highest peak intensity was observed for [THEA][Ole], followed by [BHEA][Ole] and [2-HEA][Ole], indicating that the number of hydroxyethyl substituents on the cation significantly influences the charge density distribution. The enhanced peak intensity exhibited by [THEA][Ole] suggests a greater degree of charge accumulation, which can be attributed to the formation of stronger hydrogen-bonding networks and reinforced electrostatic interactions arising from the increased number of hydroxyethyl groups. This systematic trend highlights the critical role of cation structural modification in governing the electronic characteristics of SAILs. In contrast, the rigid aromatic frameworks of the pharmaceutical compounds confine electron density to distinct molecular regions, resulting in sharper and more localized peaks in the *σ*-profile. Such localized charge distributions play a crucial role in determining the solubilization behavior and intermolecular interaction patterns of these drug molecules. These effects become particularly important in micellar environments, where the distribution of surface charge governs molecular recognition, drug–surfactant interactions, and the overall organization of supramolecular assemblies^17,99^.

The disparities exhibited in the *σ*-profiles bear profound ramifications for the solution-phase physicochemical attributes of these molecular entities. The expansive charge density distribution characteristic of the SAILs intimates an augmented degree of electronic delocalization, which potentiates their solvation efficacy in polar media and engenders facilitated associations with charged interfaces such as bio membranes or nanoparticle facets. This intrinsic attribute renders SAILs eminently amenable to modalities demanding amplified solubility, electrostatic mediation, or amphiphilic self-organization into micellar architectures. In juxtaposition, the circumscribed charge localization inherent to DFS portends a more delimited solvation profile, potentially modulating its propensity for micellization and the intensity of intermolecular engagements. The nexus between charge delocalization and architectural motifs is further instantiated by the sequential perturbations across the SAIL congeners. The profusion of hydroxyl appendages in [THEA][Ole] precipitates the paramount electron density summit, underscoring its superior capacity for electrostatic charge attenuation^100–102^. This phenomenology harmonizes with the empirically discerned diminution in limiting molar conductivity, wherein the intensified electron density may foster the consolidation of more resilient ion pairs, thereby attenuating ionic translational freedom. The more diffuse charge partitioning in extended-chain SAIL variants, exemplified by [BHEA][Ole] and [THEA][Ole], bespeaks heightened conformational pliancy, permitting a more homogeneous dissemination of screening charge over the molecular perimeter. Conversely, the relatively constricted charge topography in DFS, dictated by its inflexible aromatic nucleus, reinforces subdued electrostatic affinities and curtailed solubility in protic solvents. The σ-profile interrogation unveils quintessential divergences in the electronic idiosyncrasies of SAILs vis-à-vis pharmaceutical scaffolds like DFS. The accentuated charge delocalization in SAILs amplifies their solvation proclivities and associative competencies, particularly in milieus wherein electrostatic consolidation is paramount^103^. The constricted charge regime in DFS accentuates its localized electrodynamic essence, which orchestrates its solubilization dynamics and non-covalent intermolecular couplings. These revelations furnish mechanistic perspicacity into the sub molecular deportment of these species in solvated states, while illuminating the preeminent influence of surface charge partitioning on their thermodynamic, electrostatic, and associative attributes^104^.

In computational chemistry, the properties listed in Table 3 provide critical insights into the structural and electronic characteristics of molecules, which are essential for understanding their behavior in various environments. The surface area (*A*) and cavity volume (*V*), calculated using the COSMO (Conductor-like Screening Model) method, represent the solvent-accessible surface and the volume enclosed by this surface, respectively. These parameters indicate the physical size and shape of the molecule within a solvent, influencing its solvation properties. The dielectric solvation energy encapsulates the energetic stabilization accrued by the solute upon embedding within the solvent continuum, wherein more negative magnitudes denote fortified electrostatic interactions with the solvating medium. The frontier electronic descriptors, commencing with the highest occupied molecular orbital (HOMO) and lowest unoccupied molecular orbital (LUMO) energies, furnish quintessential metrics for appraising the intrinsic reactivity of the molecular entityb (Table 8).

**Table 8:** The surface area (*A*) and total volume (*V*) of cavity, dielectric (solvation) energy, HOMO and LUMO values and energies, ionization potential (IP), electron affinity (EA), HOMO-LUMO gap (ΔE), chemical hardness (*η*), chemical softness (S), chemical potential (*µ*), and electrophilicity index (*ω*) obtained from COSMO and Dmol^3^ calculations.

Materials	A (A^2^)	V (A^3^)	Solvation energy (kcal·mol^− 1^)	*n*_HOMO_	*n*_LUMO_	E_HOMO_ (ev)	E_LUMO_ (ev)	IP (eV)	EA (eV)	ΔE (eV)	η (eV)	S (eV⁻¹)	µ (eV)	ω (eV)
Diclofenac sodium	259.21	282.5	-19.46	76	77	-4.86	-2.09	4.86	2.09	2.77	1.39	0.72	3.48	4.36
[2-HEA][Ole]	495.7	464.73	-142.57	96	97	-4.09	-0.2	4.09	0.2	3.89	1.95	0.51	2.15	1.18
[BHEA][Ole]	545.41	514.87	-136.93	108	109	-3.94	-0.14	3.94	0.14	3.8	1.90	0.53	2.04	1.10
[THEA][Ole]	584.37	565.93	-131.74	120	121	-4.28	-0.23	4.28	0.23	4.05	2.03	0.49	2.26	1.26

Frontier molecular orbital (FMO) energies are widely recognized as fundamental descriptors of molecular reactivity and electronic behavior. The energy of the highest occupied molecular orbital (HOMO), which is approximately related to the negative ionization potential (IP), reflects the propensity of a molecule to donate electrons. Conversely, the energy of the lowest unoccupied molecular orbital (LUMO), approximated as the negative electron affinity (EA), represents its ability to accept electrons. The HOMO–LUMO energy gap (ΔE), defined as the difference between the LUMO and HOMO energies, is an important indicator of molecular stability and chemical reactivity. Generally, smaller ΔE values are associated with higher reactivity, as they facilitate electron excitation and charge-transfer processes. Several conceptual density functional theory (DFT) descriptors can be derived from these orbital energies to further characterize molecular electronic properties. Among these, chemical hardness (η), expressed as η = (IP − EA)/2, measures the resistance of a molecular system to changes in its electron density. Molecules with larger hardness values are typically more electronically stable and less prone to charge redistribution, whereas lower hardness values indicate greater polarizability and enhanced chemical reactivity.

Chemical softness (S = 1/η) represents the susceptibility of a molecular system to electronic perturbation and reflects its propensity for charge redistribution. In contrast, the chemical potential (μ = −(IP + EA)/2) describes the tendency of a molecule to exchange electrons with its surroundings, while the electrophilicity index (ω = μ²/2η) quantifies its electron-accepting capability. Higher electrophilicity values indicate a greater tendency to stabilize additional electronic charge and, consequently, stronger electrophilic character. The structural and solvation parameters reveal pronounced differences between diclofenac sodium (DFS) and the investigated ammonium oleate derivatives. DFS possesses a molecular surface area of 259.21 Å² and a cavity volume of 282.50 Å³, both substantially smaller than those of the studied SAILs, which exhibit surface areas ranging from 495.70 to 584.37 Å² and cavity volumes between 464.73 and 565.93 Å³. Furthermore, the dielectric solvation energy of DFS (−19.46 kcal·mol⁻¹) is significantly less negative than those of the oleate derivatives (−142.57 to −131.74 kcal·mol⁻¹), indicating comparatively weaker stabilization by the solvent environment. These findings are consistent with the compact molecular architecture and lower polarizability of DFS relative to the larger, amphiphilic structures of the oleate-based ionic liquids. The calculated conceptual DFT descriptors further highlight the distinct electronic characteristics of the studied species. DFS exhibits HOMO and LUMO energies of −4.86 and −2.09 eV, respectively, resulting in a HOMO–LUMO energy gap of 2.77 eV. Correspondingly, the calculated ionization potential, electron affinity, chemical hardness, chemical softness, chemical potential, and electrophilicity index are 4.86 eV, 2.09 eV, 1.38 eV, 0.722 eV⁻¹, −3.47 eV, and 4.36 eV, respectively. In contrast, the ammonium oleate derivatives display HOMO energies ranging from −4.28 to −3.94 eV and LUMO energies from −0.23 to −0.14 eV, yielding larger HOMO–LUMO gaps (3.80–4.05 eV). These compounds also exhibit higher chemical hardness values (1.90–2.02 eV) and considerably lower electrophilicity indices (1.09–1.25 eV). Collectively, these results indicate that DFS is electronically more reactive and less kinetically stable than the ammonium oleate derivatives. This behavior can be attributed to its conjugated aromatic framework and the presence of electron-withdrawing substituents, which facilitate charge transfer and enhance electrophilic character. Conversely, the larger HOMO–LUMO gaps, greater hardness, and lower electrophilicity of the oleate derivatives suggest enhanced electronic stability and reduced intrinsic reactivity. These complementary electronic characteristics support the formation of favorable intermolecular interactions between DFS and the oleate-based ionic liquids, thereby providing a molecular-level rationale for the enhanced solubilization behavior observed experimentally. ^105^. These results suggest that DFS is more reactive and possesses moderate chemical stability compared to the oleate derivatives, likely attributable to its aromatic framework and functional groups. These electronic characteristics highlight DFS suitability for pharmaceutical applications where specific reactivity and stability profiles are essential^36,106,107^.

  The HOMO energy reflects the electron-donating capability of a species, whereas the LUMO energy represents its propensity to accept electrons. The calculated orbital energies suggest that the SAILs ions possess favorable electronic properties for donor–acceptor interactions with DFS, particularly through its carboxylate functionality and aromatic framework. Furthermore, the hydroxyl groups present in the 2-hydroxyethylamine-based cations are capable of facilitating hydrogen-bonding interactions, while the long hydrophobic oleate chain provides a nonpolar environment that can effectively accommodate the aromatic moieties of DFS. The synergistic contribution of these electrostatic, hydrogen-bonding, and hydrophobic interactions is expected to enhance the affinity of DFS toward the SAIL medium, thereby contributing to its improved solubilization behavior observed experimentally. It is important to note that the present COSMO-DFT analysis is intended to offer qualitative molecular-level interpretation of the observed solubility enhancement rather than a quantitative prediction of solvation thermodynamics. Nevertheless, the computational findings could provide valuable mechanistic support for the experimentally observed drug–SAILs interactions and their role in promoting DFS solubility.

Although toxicity and biocompatibility were not evaluated in the present study, the investigated SAILs are derived from 2-hydroxyethylamine and fatty‑acid‑based components that are commonly used in cosmetic and pharmaceutical formulations. Such bio‑based ionic liquids have been reported to exhibit relatively low toxicity and improved environmental compatibility compared with conventional ionic liquids, suggesting their potential suitability for pharmaceutical and formulation applications. Nevertheless, detailed cytotoxicity and biocompatibility assessments are required and will be addressed in future studies.

## Conclusion

This study presents a detailed investigation of the intermolecular interactions between diclofenac sodium (DFS) and a series of surface-active ionic liquids (SAILs), including (2-hydroxyethyl)ammonium oleate ([2-HEA][Ole]), bis(2-hydroxyethyl)ammonium oleate ([BHEA][Ole]), and tris(2-hydroxyethyl)ammonium oleate ([THEA][Ole]). Fluorescence spectroscopy measurements were performed at 298.15 K to investigate the fluorescence quenching behavior of DFS in the presence of the investigated SAILs. The results indicate that the quenching process predominantly follows a static mechanism, suggesting that the observed decrease in fluorescence intensity arises from the formation of ground-state complexes between DFS and the SAILs rather than from dynamic collisional interactions. This interpretation is further supported by the calculated Stern–Volmer quenching constants (*K*_SV_) and bimolecular quenching rate constants (*K*_q_), whose magnitudes are consistent with static quenching behavior. These findings confirm that fluorescence suppression originates primarily from stable DFS–SAIL complex formation. The binding interactions were further evaluated through the determination of the association constants (*K*_A_) and binding site parameters (*n*) for the DFS–SAIL systems. Among the investigated SAILs, [THEA][Ole] exhibited the highest binding affinity toward DFS, indicating stronger intermolecular interactions compared with [BHEA][Ole] and [2‑HEA][Ole]. This enhanced affinity may arise from favorable hydrogen‑bonding and electrostatic interactions that promote DFS–SAIL association and stabilization. Equilibrium solubility measurements of DFS in SAIL–water mixtures across varying SAIL mass fractions and temperatures (298.15 to 313.15) K showed a consistent increase in mole‑fraction solubility with both SAIL concentration and temperature, reflecting entropy contributes favorably at lower SAIL compositions, but its relative contribution decreases as SAIL concentration increases. In contrast to the binding behavior, the solubility data clearly demonstrate that overall solubilization follows the order [THEA][Ole] < [BHEA][Ole] < [2‑HEA][Ole], with [2‑HEA][Ole] providing the greatest solubilization enhancement at higher SAIL loadings.

To evaluate the non‑ideal solution thermodynamics, the experimental solubilities were correlated using the Wilson and electrolyte‑NRTL (*e*‑NRTL) models. The Wilson model provided superior agreement (ARD% ≈ 0.20) compared with *e*‑NRTL (5.34%), indicating that local‑composition models effectively capture the amphiphilic structuring within SAIL–water media. Complementarily, DFT calculations combined with COSMO solvation analysis provided molecular‑level insight into the non‑covalent interactions governing DFS–SAIL association. Assessments of cavity volumes, solvation energies, and σ‑profiles revealed distinct electronic and structural features among the SAILs, with [THEA][Ole] showing stronger electrostatic complementarity with DFS, consistent with its higher binding affinity but distinct from its lower macroscopic solubilization capacity relative to [2‑HEA][Ole]. These computational findings help rationalize the interplay between molecular‑scale interactions and bulk solubility behavior and offer a basis for designing next‑generation SAILs to enhance drug solubility and bioavailability.

## Supplementary Information

Below is the link to the electronic supplementary material.


Supplementary Material 1


## Data Availability

The authors confirm that the data supporting the findings of this study are available within the manuscript, figures, tables, and supporting information files.
